# Absence of Rapid Propagation through the Purkinje Network as a Potential Cause of Line Block in the Human Heart with Left Bundle Branch Block

**DOI:** 10.3389/fphys.2018.00056

**Published:** 2018-02-06

**Authors:** Jun-ichi Okada, Takumi Washio, Machiko Nakagawa, Masahiro Watanabe, Yoshimasa Kadooka, Taro Kariya, Hiroshi Yamashita, Yoko Yamada, Shin-ichi Momomura, Ryozo Nagai, Toshiaki Hisada, Seiryo Sugiura

**Affiliations:** ^1^Department of Human and Engineered Environmental Studies, Graduate School of Frontier Sciences, The University of Tokyo, Kashiwa, Japan; ^2^Healthcare System Unit, Fujitsu Ltd., Tokyo, Japan; ^3^Department of Cardiovascular Medicine, School of Medicine, The University of Tokyo, Tokyo, Japan; ^4^Department of Cardiovascular Medicine, Saitama Medical Center, Jichi Medical University, Saitama, Japan

**Keywords:** multi-scale simulation, conduction block, non-contact mapping, line of block, Purkinje network, electrophysiology

## Abstract

**Background:** Cardiac resynchronization therapy is an effective device therapy for heart failure patients with conduction block. However, a problem with this invasive technique is the nearly 30% of non-responders. A number of studies have reported a functional line of block of cardiac excitation propagation in responders. However, this can only be detected using non-contact endocardial mapping. Further, although the line of block is considered a sign of responders to therapy, the mechanism remains unclear.

**Methods:** Herein, we created two patient-specific heart models with conduction block and simulated the propagation of excitation based on a cellmodel of electrophysiology. In one model with a relatively narrow QRS width (176 ms), we modeled the Purkinje network using a thin endocardial layer with rapid conduction. To reproduce a wider QRS complex (200 ms) in the second model, we eliminated the Purkinje network, and we simulated the endocardial mapping by solving the inverse problem according to the actual mapping system.

**Results:** We successfully observed the line of block using non-contact mapping in the model without the rapid propagation of excitation through the Purkinje network, although the excitation in the wall propagated smoothly. This model of slow conduction also reproduced the characteristic properties of the line of block, including dense isochronal lines and fractionated local electrocardiograms. Further, simulation of ventricular pacing from the lateral wall shifted the location of the line of block. By contrast, in the model with the Purkinje network, propagation of excitation in the endocardial map faithfully followed the actual propagation in the wall, without showing the line of block. Finally, switching the mode of propagation between the two models completely reversed these findings.

**Conclusions:** Our simulation data suggest that the absence of rapid propagation of excitation through the Purkinje network is the major cause of the functional line of block recorded by non-contact endocardial mapping. The line of block can be used to identify responders as these patients loose rapid propagation through the Purkinje network.

## Introduction

Although the therapeutic effects of cardiac resynchronization therapy (CRT) for patients with heart failure with conduction block have been confirmed by number of clinical trials, the existence of non-responders remains a major health and economic problem (Kirk and Kass, 2013; Prinzen et al., 2013; Yu and Hayes, 2013). Accordingly, there are numerous studies examining the mechanisms leading to electromechanical dyssynchrony in affected patients, to aid in the identification of individuals responding to treatment (Bax et al., 2005a,b). Indeed, electrophysiological studies have shown that a wide QRS on surface ECG, reflecting a long delay in propagation of activation, is a reliable marker of responders (Auricchio et al., 2003; Brignole et al., 2013). However, in a detailed analysis of the activation sequence in the left ventricle (LV) using the unipolar non-contact mapping system to assess the activation wave front, Auricchio et al. reported that the LV activation sequence in patients with heart failure and left bundle-branch block was blocked by a narrow region of slow conduction (line of block), which could not cross directly to the lateral wall from the anterior region (Auricchio et al., 2004). Similar observations were also reported, and the line of block was suggested as a biomarker of the response to CRT (Fung et al., 2004; Lambiase et al., 2004; Duckett et al., 2012). Nevertheless, bipolar contact mapping of the LV endocardium performed in the same patients revealed a continuous propagation of the activation wave front, and were thus unable to demonstrate a line of block (Auricchio et al., 2004). Unipolar signals by non-contact mapping were recorded by 64 electrodes mounted equidistantly on a 7.6 mL balloon (size 1.8 × 4.6 cm) placed in the LV cavity. From these signals, endocardial mapping was generated by solving the inverse problem using the boundary element method. An optimized regularization was required to produce accurate results, and a spline curve fit was used to produce 3,360 potential points on the endocardial surface. However, as the raw signals recorded in the LV cavity were far-field electrographic data, the generated non-contact endocardial map retains electrical information from the transmural thickness (Gornick et al., 1999). By contrast, the bipolar signals, recorded by pushing a pair of leads separated by 2 or 5 mm on the endocardial surface, represent the local information from the endocardium. The mapping procedure was performed for approximately 200 contact points equally distributed on the LV endocardial surface. Accordingly, the authors speculated that the line of block reflects the abnormal propagation pattern of intramural activation (Auricchio et al., 2004). However, this underlying mechanism has not been confirmed clinically.

Computer simulations are an indispensable tool for studying cardiac pathophysiology (Noble, 2002; Winslow et al., 2012). In particular, multi-scale simulations using a realistic three-dimensional heart model provide an opportunity to examine the normal and abnormal cardiac functions resulting from the complex interplay of molecular, cellular, and tissue-level activity. We also developed a multi-scale heart simulator (Watanabe et al., 2004; Washio et al., 2010, 2013; Sugiura et al., 2012), and reported its various applications including patient-specific electrophysiology of normal and diseased hearts (Okada et al., 2011, 2013), prediction of drug-induced arrhythmogenic risk (Okada et al., 2015), and CRT (Panthee et al., 2016; Okada et al., 2017). In our previous study, using CRT in patient-specific models of failing hearts with conduction block (Okada et al., 2017), we examined the intramural activation sequences of variably affected heart models, and found that either the absence or presence of the rapid propagation of activation through the Purkinje system in the endocardium determines the QRS width in the ECG of patients with left bundle branch block. We hypothesize that this difference in activation pattern within the ventricular wall may be also the cause of the line of block observed by non-contact mapping in left bundle-branch cases.

To test this hypothesis, in the present study we simulated non-contact mapping in our diseased heart models. We demonstrated that the slow propagation of activation without the involvement of the Purkinje system was recorded and processed by the algorithm of the non-contact mapping system to produce the line of block, thus supporting our hypothesis. This observation promotes our understanding on the pathophysiology of left bundle-branch block, and also characterizes the information retrievable from non-contact mapping of the heart.

## Materials and methods

In this study, we used a number of models of heart failure with left bundle branch block using clinical data collected with written informed consent after approval by the institutional review board, as previously reported (Okada et al., 2017).

### Multi-scale simulation of the cardiac excitation and propagation

**Figure 1:**
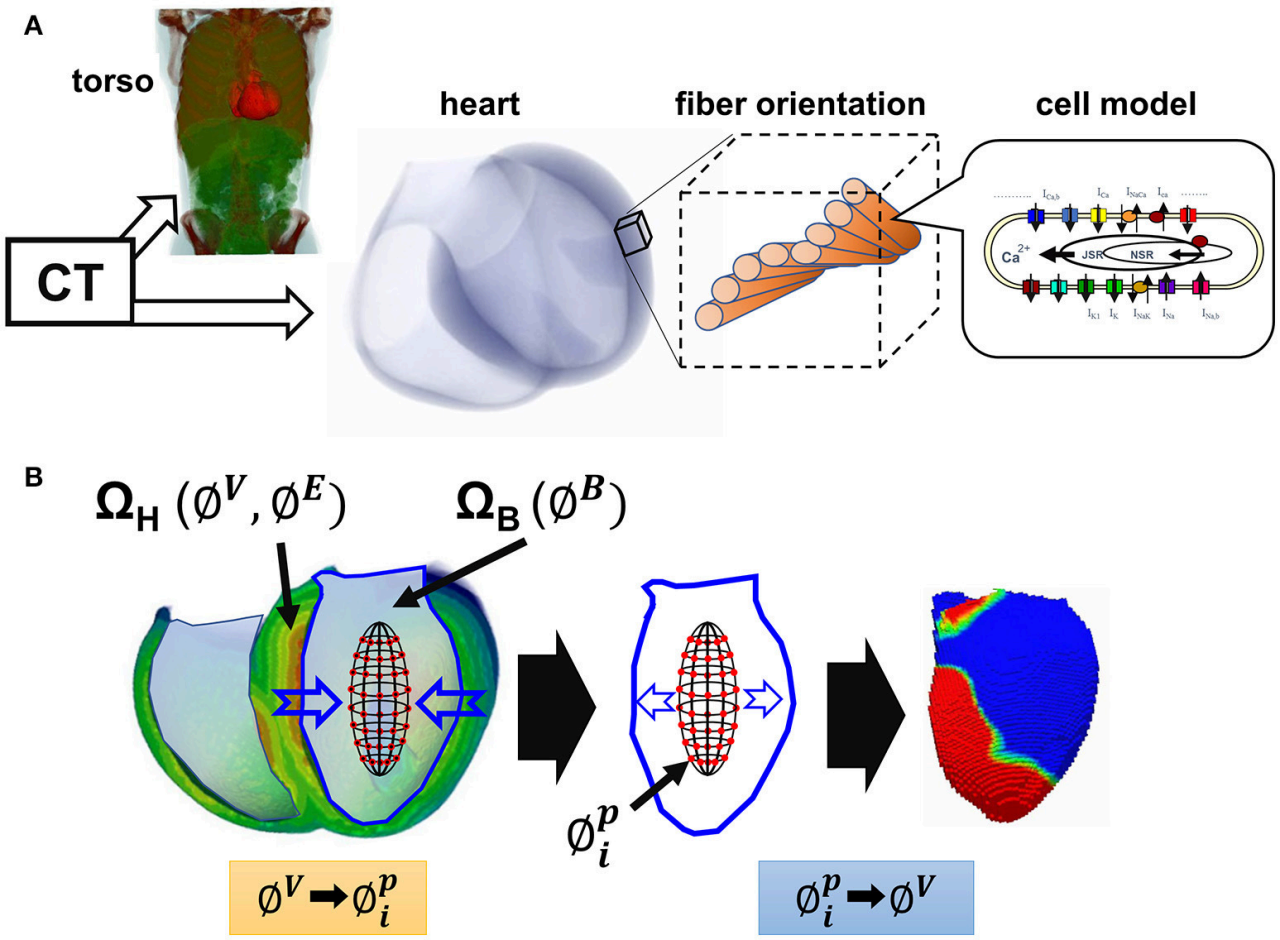
Multi-scale simulation of cardiac excitation and propagation. **(A)** Schematic presentation of the multi-scale heart model. **(B)** Simulation of non-contact endocardial mapping. Ω_H_, heart wall domain; Ω_B_, ventricular cavity domain; ∅^*V*^, membrane potential; ∅^*E*^, potential in the extracellular space; ∅^*B*^, potential in the cavity; $\emptyset_{i}^{p}$, *I* = 1 to 64: potential recorded by the 64 electrode array.

Details of the multi-scale, multi-physics heart simulator were previously reported (Watanabe et al., 2004; Washio et al., 2010, 2013; Okada et al., 2011, 2013, 2015; Sugiura et al., 2012). In the present study, the simulation of electrophysiology was performed using only the finite element models of the ventricles and torso (Okada et al., 2017). In brief, the voxel model of the heart (mesh size: 0.4 mm, number of elements: 32,768,000 and 36,126,720) and the torso (mesh size: 1.6 mm) were created from the three dimensional reconstruction of multi-slice computed tomography data. In the heart model, we mapped the fiber orientation using our rule-based method (Hisada et al., 2013), so that the fiber orientations changed across the wall depth from the endocardium to the epicardium (LV free wall: −90° to 60°, interventricular septum: −90° to 70°, RV wall: −60° to 60°). To each element, we implemented the human ventricular myocyte model of electrophysiology (Ten Tusscher et al., 2004), with three cell types with different action potential duration; i.e., endocardial, M-, and epicardial cells. The distribution of these sub-types of cells was similar to that we previously reported, in which M-cells were located at 10–40% from the endocardial surface (Okada et al., 2011). The conduction system was modeled by a thin layer on the surface of the endocardial region (thickness 0.8 mm) with a high conduction velocity (1.7 m/s (default)). For the simulation of sinus rhythm, we applied small currents to excite the earliest activation sites identified to reproduce the previously reported isochronal maps (Durrer et al., 1970) and body surface voltage maps (Taccardi, 1963; De Ambroggi et al., 1976; Figure 1A). Propagation of excitation in the heart domain was analyzed by solving the following bidomain equation.

$$\begin{array}{l} \beta \left(C_{m}\frac{\partial V}{\partial t}+I_{ion}\right)=I_{stim}-\frac{\partial }{\partial x_{i}}\left(G_{ij}^{E}\frac{\partial \emptyset^{E}}{\partial x_{j}}\right) \end{array}$$

$$\begin{array}{l} \beta \left(C_{m}\frac{\partial V}{\partial t}+I_{ion}\right)=I_{stim}+\frac{\partial }{\partial x_{i}}\left(G_{ij}^{I}\frac{\partial \emptyset^{I}}{\partial x_{j}}\right), \end{array}$$

where ∅^*E*^ and ∅^*I*^ are the extracellular and intracellular potentials, respectively, V = ∅^*I*^ − ∅^*E*^ is the transmembrane voltage, β is the surface-to-volume ratio of the tissue, *C*_*m*_ is the membrane capacitance, t is time, $G_{ij}^{E}$ and $G_{ij}^{I}$ are the intra—and extracellular anisotropic conductivity tensors, respectively, *x*_*i*_ and *x*_*j*_ are the tensor notations of the x, y, and z coordinates, *I*_*stim*_ is the stimulation current, and *I*_*ion*_ is the sum of ionic transmembrane currents calculated by the ventricular myocyte model of electrophysiology. In the torso domain, the mono-domain equation was solved as follows.

$$\begin{array}{l} -\frac{\partial }{\partial x_{i}}\left(G_{ij}\frac{\partial \emptyset }{\partial x_{j}}\right)=0, \end{array}$$

where ∅ is the potential and *G*_*ij*_ is the anisotropic conductivity at each point. Detailed information on numerical methods can be seen in Supplementary Material. Simulations were performed for a heart rate of 1 Hz using only the ventricles. Default conductivity values used for the heart and torso were taken from reported literature (Camacho et al., 1995; Panescu et al., 1995; Keldermann et al., 2009) and are listed in Table 1. To reproduce the clinical ECG, we adjusted the conductivity for each patient.

**Table 1:** Conductivity values used in the model.

**Tissue**	**Conductivity (mS/cm)**
**HEART (INTRACELLULAR)**	
Fiber direction	6.2
Fiber normal direction)	2.0
**HEART (EXTRACELLULAR)**	
Fiber direction	4.0
Fiber normal direction)	2.0
Purkinje fiber	30.0
Blood	7.8
Atrium, artery, vein	7.0
Muscle	2.56
Lung	0.83
Esophagus, stomach, bowel	2.0
Spleen, liver, kidney	1.67
Bone	0.1
Fat	0.5
Skin, body surface	80.0

### Simulation model of heart failure with left bundle-branch block model

We used two heart models. The model with the Purkinje layer was patient 8, and the model without the Purkinje layer was patient 3, from our previous study (Okada et al., 2017). In that study, the multi-scale heart model was created according to the method described above, and we introduced left bundle-branch block by allowing only the earliest activation sites in the right ventricle. However, after the left ventricular breakthrough, we needed to adopt either of two different modes of conduction, depending on the electrophysiological properties of the patients. While slow and homogeneous conduction in the left ventricular wall reproduced the clinical ECGs of patients exhibiting a wide QRS complex, we needed to account for rapid antidromic conduction through the Purkinje system in patients with a relatively narrow QRS complex. The selection of the conduction mode was dependent on the degree of agreement between the simulated and clinical ECGs. In general, the rapid conduction mode was applied for cases with a QRS width <150 ms.

### Simulation of non-contact mapping

We simulated the unipolar non-contact mapping of the LV endocardium using a 64-electrode array, as used clinically (Gornick et al., 1999; Wang et al., 2013). The 64 electrodes were arranged concentrically and equidistantly around the surface of a prolate ellipsoid balloon (3.5 × 4.5 cm size). Details of the algorithm for obtaining the endocardial potential map are shown in Supplementary Material. In brief, we first solved the bidomain equations of the electrophysiology data in the domain consisting of the heart wall (Ω_H_) and blood inside the ventricular cavity containing blood (Ω_B_). From this we obtained the membrane potential (∅^*V*^), and the potential in the extracellular space (∅^*E*^) in Ω_H_ and the potential in Ω_B_ (∅^*B*^) (Figure 1B, left). Potentials recorded by the 64 electrodes ($\emptyset_{1}^{p}$, *i* = 1 to 64) were sampled at the corresponding position in the blood domain, and were expressed by the following matrix equation.

$$\begin{array}{l} \emptyset^{p}=\left(\begin{array}{c} \emptyset_{1}^{p}\\ \vdots \\ \emptyset_{64}^{p} \end{array}\right)=B\cdot \left(\begin{array}{c} \emptyset_{1}^{H}\\ \vdots \\ \emptyset_{N}^{H} \end{array}\right)=B\cdot \emptyset^{H}, \end{array}$$

where N is the number of nodes in the heart domain. It is important to note that these potentials recorded by the electrode array reflected the electrical activity across the whole transmural thickness, not the mirror image of the endocardial potential. Finally, endocardial potentials were obtained as the inverse solution (Figure 1B, middle and right). However, as this is an ill-posed problem, we solved an optimization problem by minimizing the following function (Wang et al., 2013).

$$\begin{array}{l} E\left(\emptyset^{H}\right)=\frac{1}{2}∥B\cdot \emptyset^{H}-\emptyset^{p}{∥}^{2}+\frac{\varepsilon }{2}\left(\emptyset^{H}\cdot L\cdot \emptyset^{H}\right), \end{array}$$

where *L* is the Laplacian operator. The second term on the right-hand side is a regularization term for stabilizing the minimization.

### Computation

All program code was written in FORTRAN in our laboratory. Simulations were performed using a 2.6-GHz HP Blade System c7000 (Intel Xeon E5-2670; Intel, Santa Clara, CA, USA). With the total number of degrees of freedom set to approximately 60 million, it took 50 min to compute a single cardiac cycle using 127 cores. Numerical data were visualized using commercial software (MicroAVS; Advanced Visual Systems, Waltham, MA, USA).

## Results

### Slow propagation excluding the conduction system introduces the line of block

**Figure 2:**
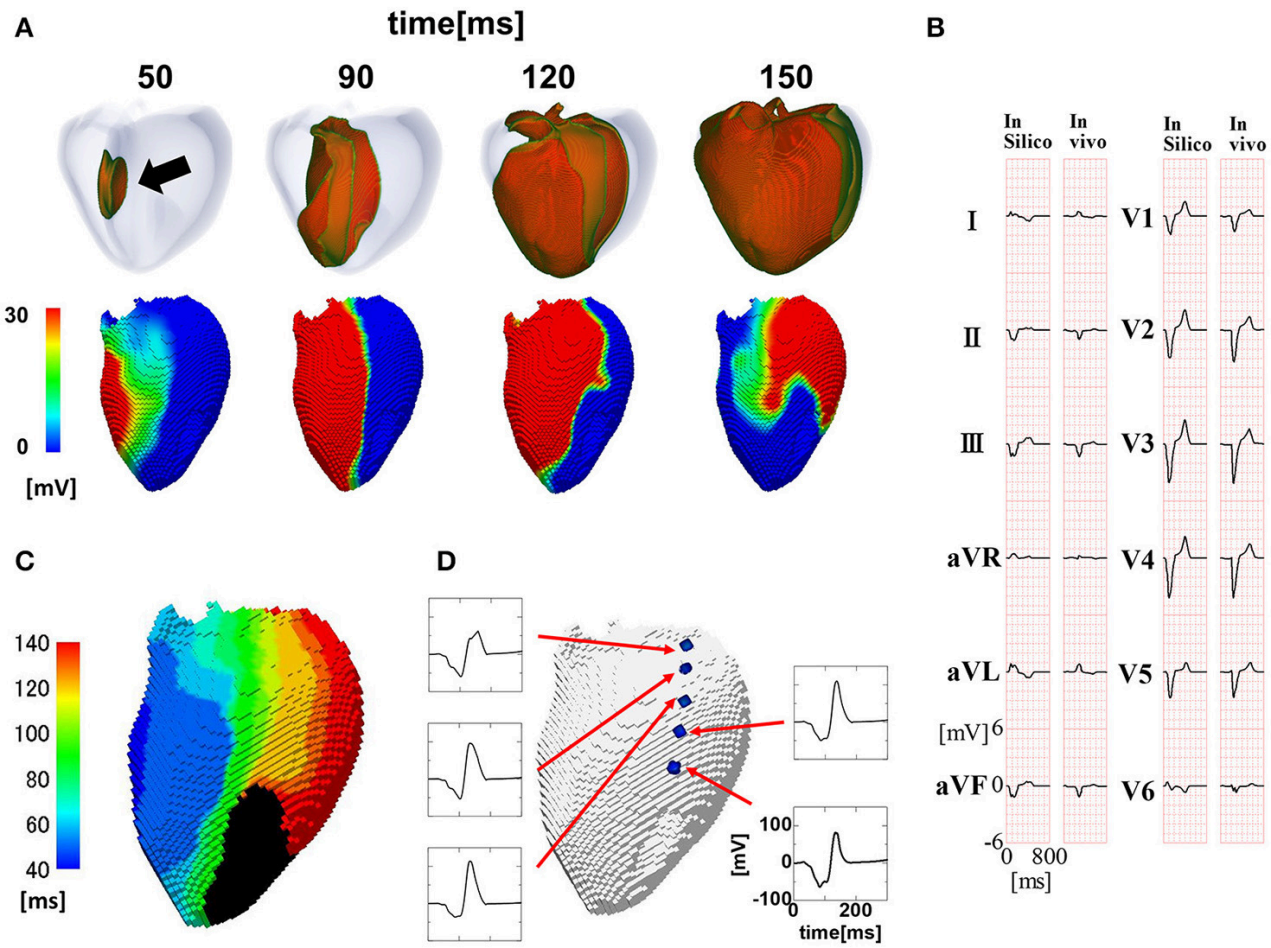
Propagation of excitation in the heart with a narrow QRS width. **(A)** Time-lapse images of the activation in the ventricular wall (upper row: Region of ventricular wall where membrane voltage exceeds −50 mV is shown in color) and corresponding non-contact endocardial map (lower row). Numbers at the top indicate the time after the activation starts. Black arrow: left ventricular breakthrough; color bar: membrane potential. **(B)** Real (left column) and simulated (right column) 12 lead body surface electrocardiogram (ECG). **(C)** Color-coded isochronal map (left) and local unipolar electrocardiogram. **(D)** Local unipolar electrocardiogram.

The activation sequence in the entire ventricular wall (upper row) and the results of endocardial non-contact mapping (lower row) in the patient with a relatively narrow QRS complex (176 ms Figure 2B) are shown in Figure 2A (see also Supplemental Movie S1). During activation of the entire wall (upper row), activation of endocardial conduction system clearly preceded that of the outer layer, while the activation pattern of the non-contact mapping (lower row) smoothly followed that of the conduction system. In this case, we could not identify the line of block in the endocardial isochronal map (Figure 2C) or the fractionated regional electrocardiogram (Figure 2D).

**Figure 3:**
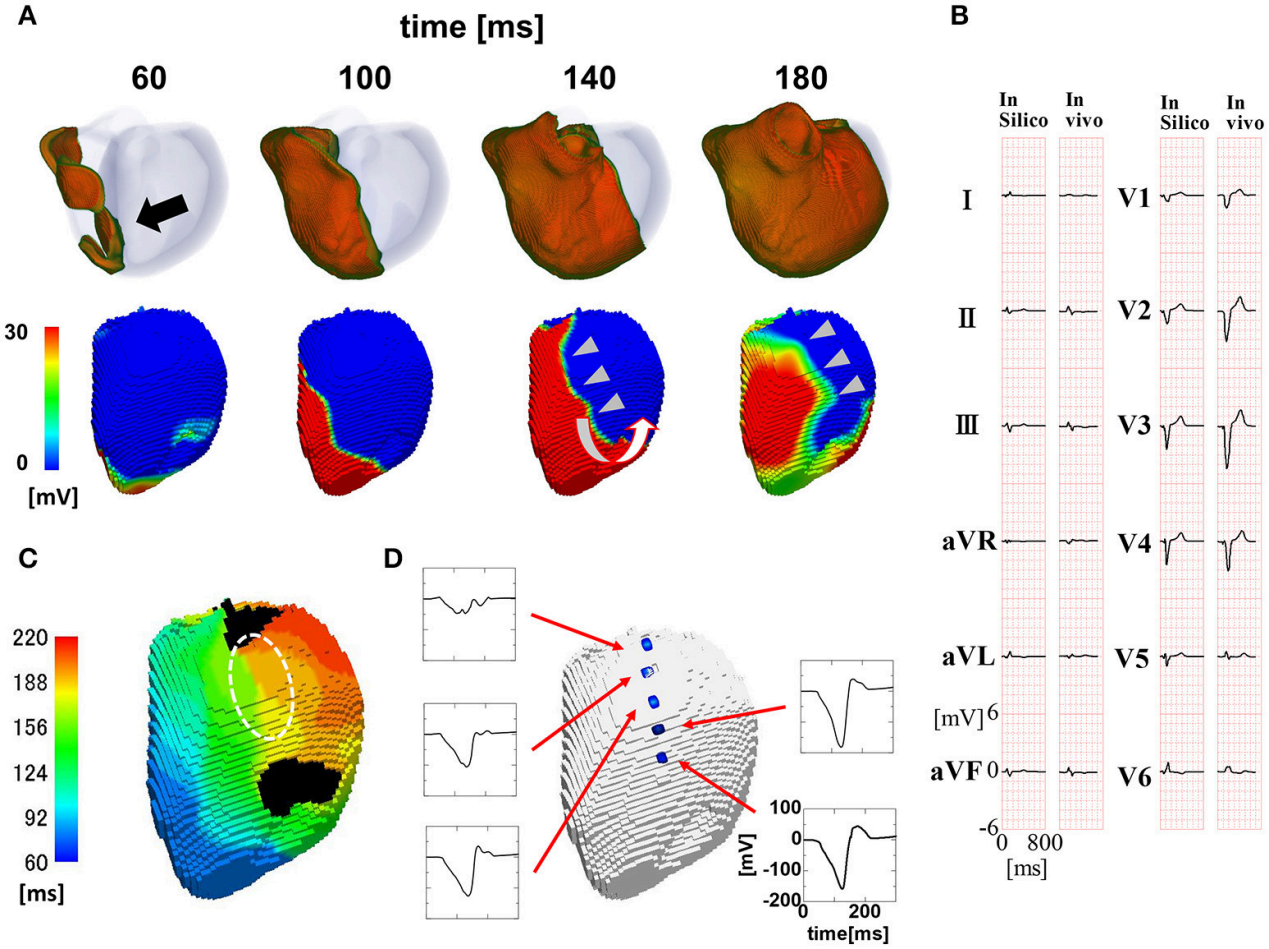
Propagation of excitation in the heart with a wide QRS width. **(A)** Time-lapse images of the activation in the ventricular wall (upper row Region of ventricular wall where membrane voltage exceeds −50 mV is shown in color) and corresponding non-contact endocardial map (lower row). Numbers at the top indicate the time after the activation starts. Black arrow, left ventricular breakthrough; white U-shaped arrow, activation sequence; color bar, membrane potential. **(B)** Real (left column) and simulated (right column) 12 lead body surface ECG. **(C)** Color-coded isochronal map (left) and local unipolar electrocardiogram. White dotted circle indicates the region of dense isochronal lines. **(D)** Local unipolar electrocardiogram.

To reproduce the ECG with a wide QRS complex (200 ms; Figure 3B), we inhibited rapid conduction by omitting through the Purkinje network. In this case, the activation wave front started from the breakthrough point (black arrow), spanned the entire wall, and proceeded homogeneously to the free wall (Figure 3A, top row). However, the endocardial activation wave front stopped in the anterior region and turned around at the apex, thus showing a characteristic U-shaped pattern of activation sequence (white U-shaped arrow in Figure 3A, lower row) (see also Supplemental Movie S2). The isochronal map showed a region of dense isochronal lines (i.e., the line of block) at the base (white dotted circle in Figure 3C) We also identified fractionated local ECGs in the corresponding region, whereas the smooth local ECGs were observed in the apex (Figure 3D).

### Simulation model reproduced the characteristics of the functional line of block

**Figure 4:**
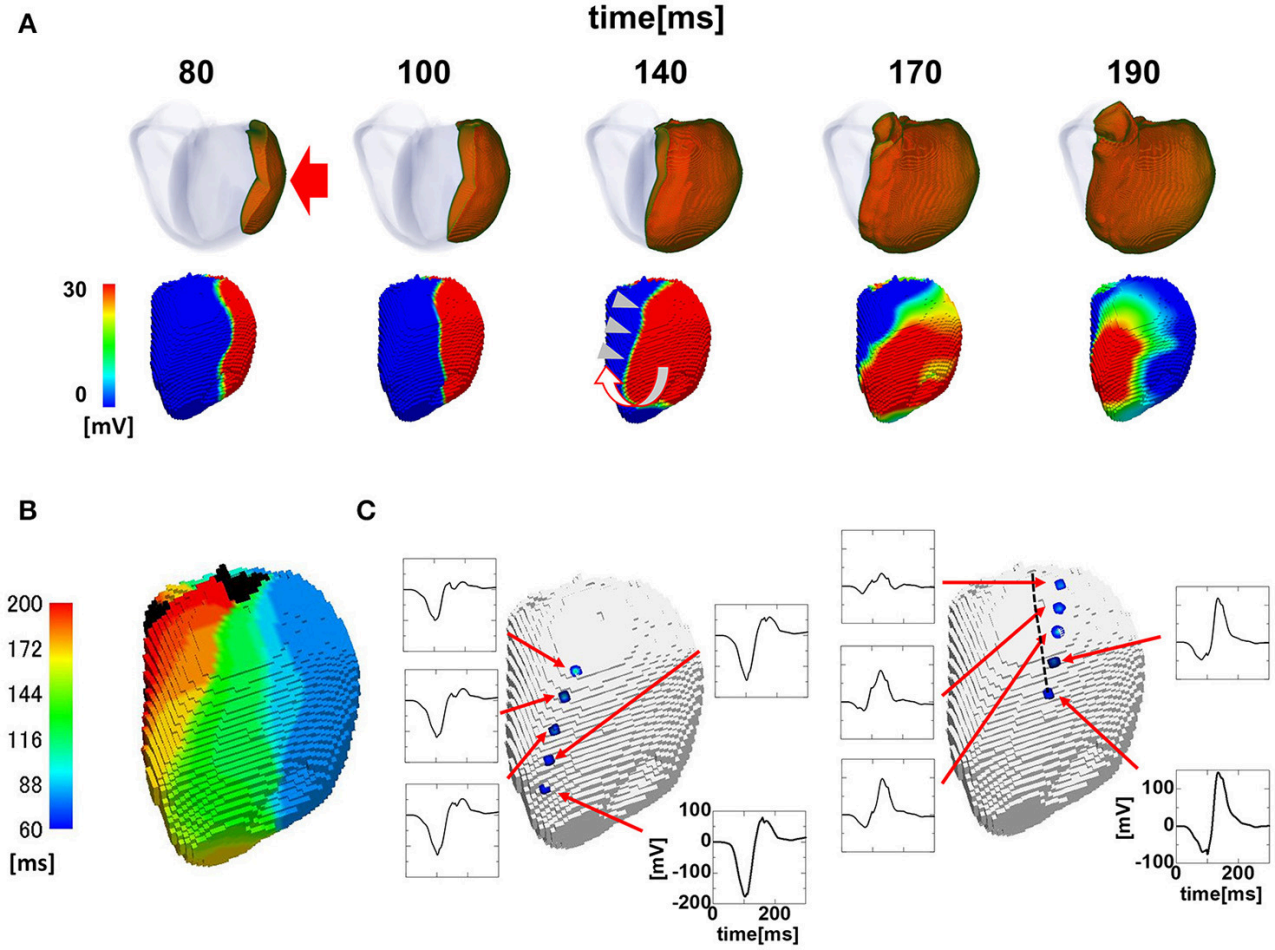
Propagation of excitation in the paced heart with a wide QRS width. **(A)** Time-lapse images of the activation in the ventricular wall (upper row) and the corresponding non-contact endocardial map (lower row). Numbers at the top indicate the time after the activation starts. Red arrow: pacing site; white U-shaped arrow: activation sequence; color bar: membrane potential. **(B)** Isochronal map. **(C)** Local unipolar ECG near the line of block. The location of the line of block under the intrinsic rhythm (Figure 3) is shown by the black broken line.

Auricchio et al. reported several findings supporting the functional nature of the line of block (Auricchio et al., 2004). We tested whether the current model of the line of block reproduced these findings. First, to determine whether the line of block in the left bundle branch block shifts from the left ventricular breakthrough point under intrinsic rhythm during pacing, we simulated the left ventricular pacing from the lateral wall (Figure 4A, red arrow). As shown in Figure 4A and the isochronal map (Figures 4B,C), the line of block shifted from the original location under pacing from the lateral wall (see also Supplemental Movie S3). The fractionated electrocardiograms identified in the region of slow conduction under intrinsic rhythm also disappeared during pacing (Figure 4C, broken line). Further, the amplitudes of both the unipolar and bipolar ECG in the region surrounding the line of block were similar to those in remote regions (i.e., near-normal; Figure 4C). Finally, the absence of scar tissue in the model of dilated cardiomyopathy excluded the possibility of morphologically-based conduction delay and block. Overall, these findings support that our simulation model reasonably reproduced the characteristics of the functional line of block observed in clinical studies (Auricchio et al., 2004; Fung et al., 2004, 2007).

### Absence of rapid conduction through the purkinje network as the predominant cause of line of block

**Figure 5:**
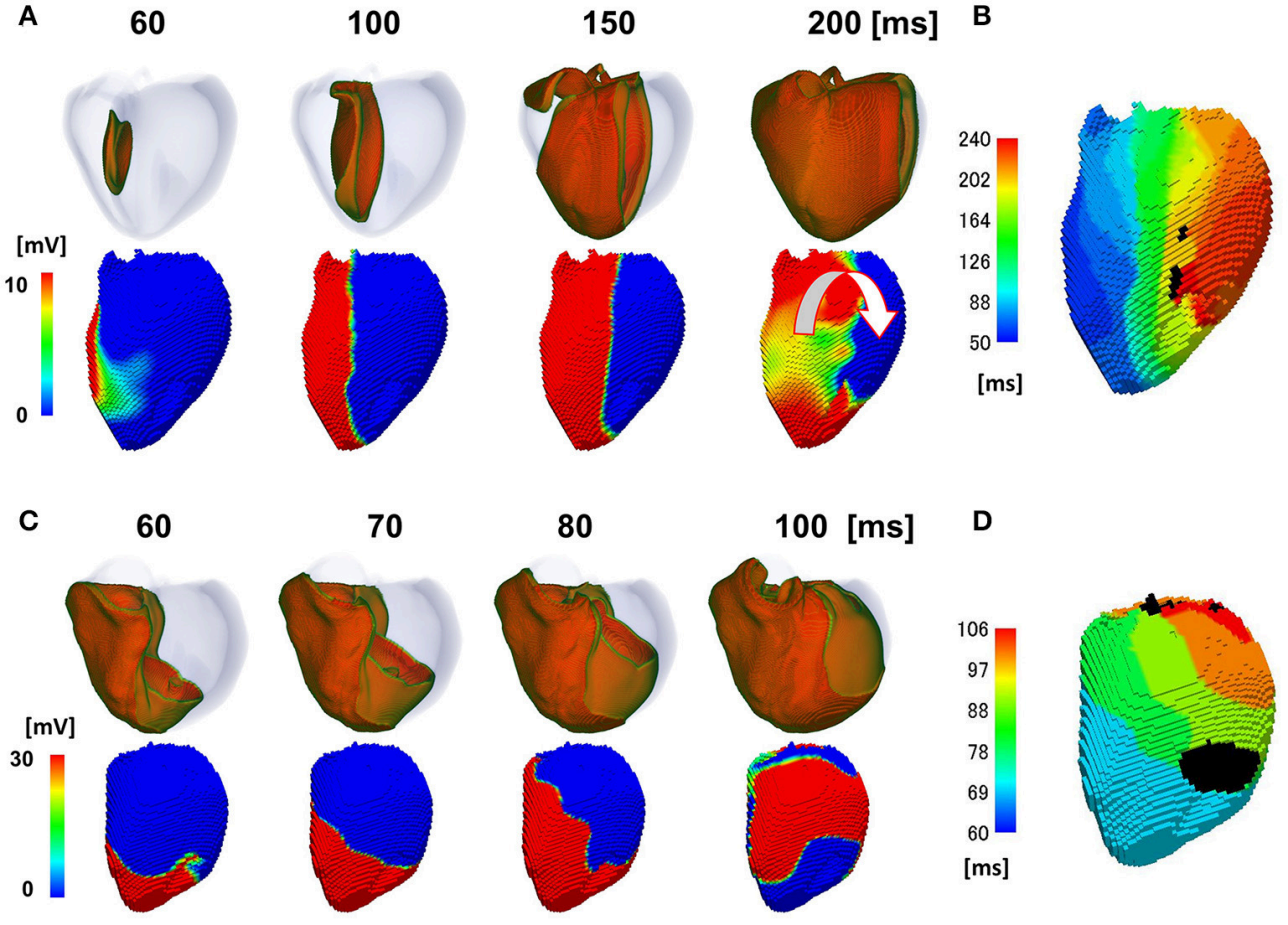
Effects of switching the mode of conduction. In the model shown in **(A**,**B)**, rapid propagation through the Purkinje network was eliminated from the model in Figure 2. **(A)** Excitation of propagation in the wall (upper row) and non-contact mapping (lower row). White U-shaped arrow: activation pattern. **(B)** Isochronal map. In the model shown in **C,D**, rapid propagation through the Purkinje network was introduced to the model in Figure 3. **(C)** Excitation of propagation in the wall (upper row) and non-contact mapping (lower row). **(D)** Isochronal map. Color-code in the non-contact maps **(A**,**C)** indicates membrane potential, while color-code in the isochronal maps **(B**,**D)** indicates the time after the activation starts (black: root of papillary muscle).

The activation sequence in the ventricle can be modified by multiple factors, including the electrophysiological property of myocytes, anisotropic conduction properties of tissue, and gross morphology of the ventricle. Our multi-scale heart simulator reproduces these properties, some of which can be personalized. To exclude the possibility that these confounding factors may contribute to the genesis of the line of block, we switched the mode of conduction between the two models shown in Figures 2, 3, and then repeated the simulations. When the rapid conduction through the endocardial Purkinje network was prevented in the model in Figure 2 by omitting the network, the QRS width increased from 176 to 238 ms, and the line of block appeared in the anterior region of the endocardial surface. The activation wave front stopped at this line and turned around to show a U-shaped pattern of activation sequence (Figures 5A,B) (see also Supplemental Movie S4). By contrast, introduction of rapid conduction through the Purkinje network eliminated the line of block (Figures 5C,D) from the model in Figure 3 (see also Supplemental Movie S5). These data suggest that the absence of rapid conduction through the Purkinje network is the predominant cause of the functional line of block observed in endocardial non-contact mapping.

## Discussion

### Functional characteristics of line of block

As unipolar non-contact mapping system can measure the global pattern of the left ventricular activation process, it has been previously used to assess the pathophysiology of heart failure patients with electromechanical abnormalities (Auricchio et al., 2004; Fung et al., 2004, 2007; Lambiase et al., 2004). For example, Fung et al. reported two patterns of activation sequence (type I and II) in heart failure patients with left bundle branch block by non-contact mapping, and patients with the type II pattern exhibiting conduction block had a more favorable response to CRT (Fung et al., 2004, 2007). The nature of conduction block was further investigated by Auricchio et al., with simultaneous application of contact and non-contact mappings (Auricchio et al., 2004). In that study, 23 out of 24 patients exhibited a U-shaped activation wave front turning around the line of block. Interestingly, however, the activation wave front proceeded smoothly by crossing the line of block in the simultaneous contact mapping. Based on the facts that (1) ventricular pacing from the coronary sinus or right ventricular apex shifted the line of block, (2) ischemic myocardial scarring was absent in two thirds of the studied patients, and (3) the amplitude of the local ECG was normal in the region surrounding the line of block, the authors concluded that the line of block identified by unipolar non-contact mapping was functional, and did not originate from the fixed scar tissue. As signals from unipolar non-contact mapping retain electrical information across the whole transmural thickness, in contrast to the bipolar contact mapping representing local electrical events, it was hypothesized that the functional line of block was caused by anisotropic conduction resulting from variation in alignment between the myocardial layer in the ventricular wall. However, there are no prior studies that have confirmed the origin of the functional line of block.

### Line of block by the simulation of non-contact mapping

We previously developed a multi-scale heart simulator in which the function and structure of the heart are reproduced at the microscopic to macroscopic levels (Sugiura et al., 2012; Washio et al., 2013). Using this technology, we also created tailor-made models of the failing heart with conduction block, with successful prediction of the responses to CRT (Okada et al., 2013, 2017; Panthee et al., 2016). Using the tailor-made failing heart models, in the present study we simulated non-contact mapping and found that the slow and homogeneous propagation of activation in the ventricular wall can cause the characteristic U-shaped activation wave front with a line of block. The presence of fragmented local ECG in the region of slow conduction, the shift of the line of block by pacing, and the disappearance of the line of block by the introduction of rapid propagation via the Purkinje network provide further support that our model reproduces the clinical observations. Overall, these findings support our hypothesis that the absence of rapid propagation via the conduction system is the major cause of the line of block in heart failure patients with conduction block.

### Potential mechanisms of line of block

**Figure 6:**
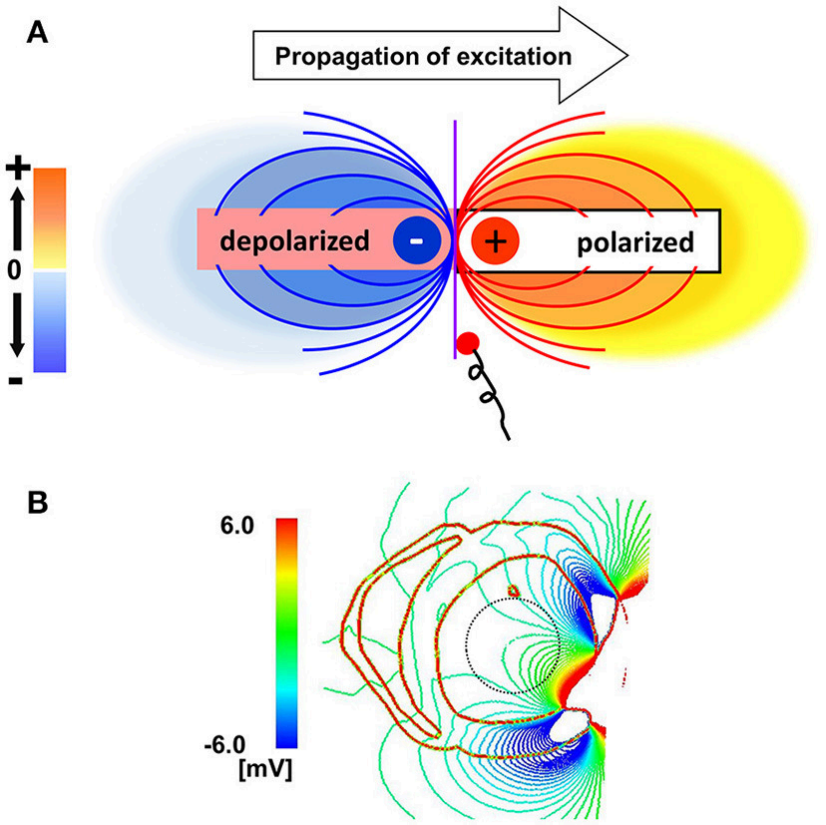
Mechanisms of the functional line of block. **(A)** Extracellular field potential generated by the activated cardiac tissue. Purple line indicates the plane of the activation front. **(B)** Extracellular field potential in a realistic ventricular morphology.

**Figure 7:**
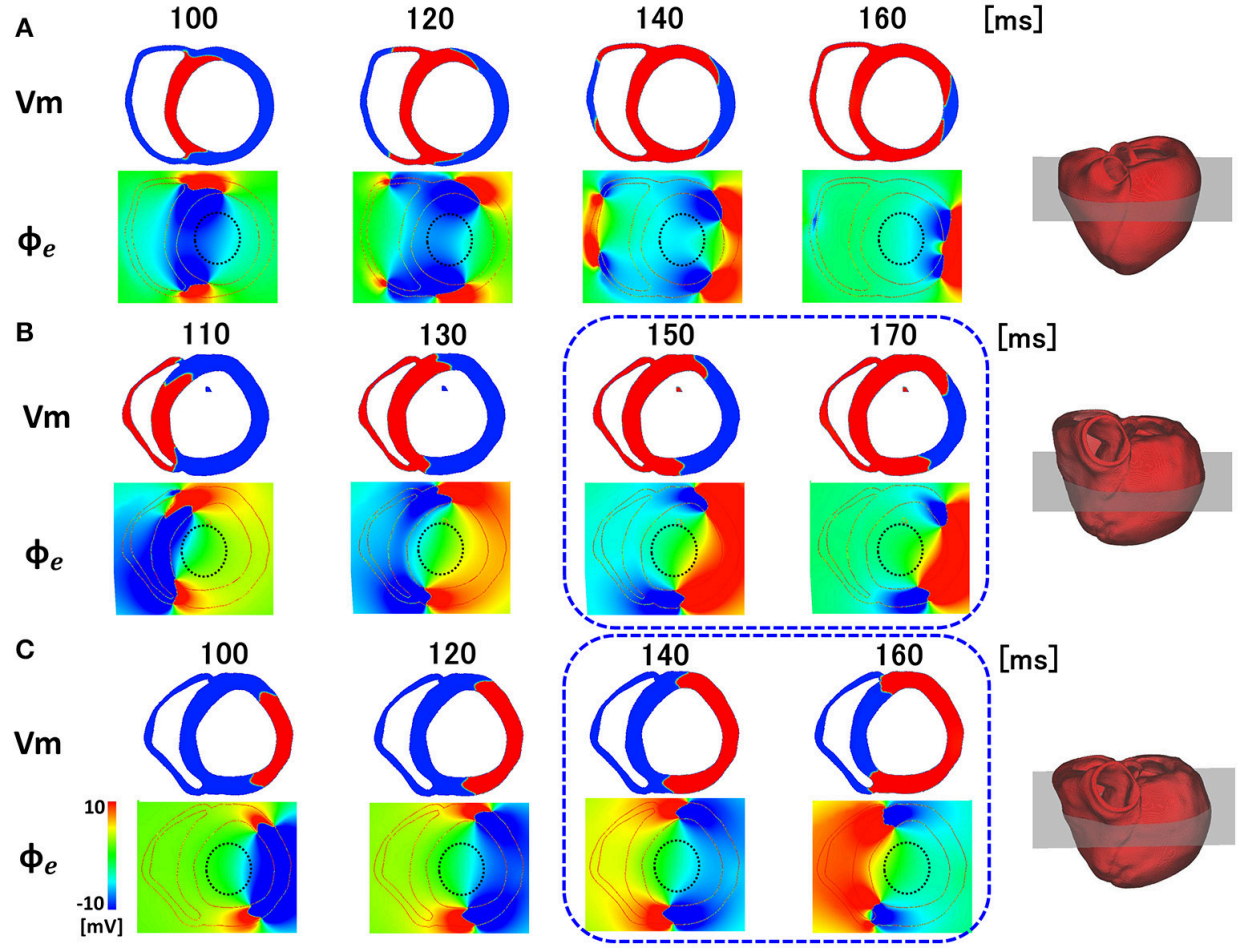
Membrane potential (V_m_) and extracellular potential field (∅_*e*_) in the various patterns of activation sequences. **(A)** Rapid propagation through the Purkinje network. **(B)** Slow propagation without the Purkinje network under the intrinsic rhythm. **(C)** Slow propagation without the Purkinje network under pacing rhythm.

What then, is the underlying mechanisms of the line of block only observed by non-contact mapping, and why does it appears in a specific position? As shown in Figure 6A, activated muscle tissue creates a field potential in the extra-cellular space centered around the activation wave front, which is measured by the non-contact electrodes. The field potential is symmetrical to the plane of the activation front (purple line), and if the electrode is placed near the plane of activation front, the electrode cannot sense the potential signal, despite its close proximity to the muscle tissue (red dot). In the real ventricle, the extracellular field potential varies in a complex manner depending on the tissue geometry and the activation sequence (Figure 6B). If the excitation propagates rapidly through the Purkinje network, the activation wave front always faces toward the epicardial surface (Figure 7A, top row), and the extracellular potential field efficiently covers the cavity throughout the ventricular activation (Figure 7A, bottom row). In this case, the basket electrode for non-contact mapping (dotted circle in the cavity) records the stable signal continuously, and this accurately reports the activation sequence. The activation sequence without rapid propagation through the Purkinje network under intrinsic rhythm is shown in Figure 7B. In this case, the activation wave front proceeds circumferentially in the wall (Figure 7B, top row), and the basket electrode cannot sense the potential field (Figure 7B, bottom row, dotted blue circle) during the ventricular activation. Under such conditions, the endocardial activation signal using non-contact mapping stalls, as the solution of the inverse problem cannot be obtained.

When the same heart was paced from the LV free wall, apparently symmetrical patterns were observed in both the activation sequence and extracellular field potential (Figure 7C). However, the timing and location of the line of block are different (blue rectangle), because of the deviation of ventricular geometry from a perfect circle. The heterogeneity of the propagation velocity in the ventricular wall, reflecting the variation in fiber orientation, is also a determinant of the location of the line of block. By contrast, in the apical portion of the ventricle with a small radius, the extracellular potential covers the cavity, even under conditions in Figures 7B,C. Accordingly, endocardial activation using non-contact mapping proceeds smoothly, and shows a U-shaped pattern of activation sequence.

### Clinical implications

If our proposed mechanism is correct, the line of block may be simply an artifact. Nevertheless, the line of block provides an indication of slow conduction occurring outside of the endocardial Purkinje network, consistent with a prior clinical report (Fung et al., 2007), thus providing an indication of favorable response to CRT. Further, the line of block provides evidence for heterogeneous conduction within the ventricular wall.

## Limitations

Although the heart models used in this study were based on clinical data, endocardial mapping was not performed in any of the patients. Thus, direct comparisons with endocardial mapping data should be performed in future studies to confirm our findings. The Purkinje network was modeled as an endocardial layer with a rapid propagation velocity and the distributions of the earliest activation sites, instead of explicitly modeling it. Although the good agreement with clinical ECG data confirmed in our previous study may rationalize this approach (Okada et al., 2013, 2017), future studies using more detailed models with the Purkinje network may provide further insight into the mechanism of the line of block in diseased hearts with conduction block.

## Author contributions

JO, TH, and SS designed the study analyzed data and wrote the paper. JO and TW wrote the programs. MN, MW, and YK performed data processing. TK, HY, YY, SM, and RN collected data and were involved in study design.

### Conflict of interest statement

JO, TW, TH, and SS have received grant support from Fujitsu Ltd. MW, YK, and MN was employed by company Fujitsu Ltd. The other authors declare that the research was conducted in the absence of any commercial or financial relationships that could be construed as a potential conflict of interest.
