# Supplementary material for: Absence of Rapid Propagation through the Purkinje Network as a Potential Cause of Line Block in the Human Heart with Left Bundle Branch Block
Source: Front Physiol. 2018 Feb 6;9:56. doi: 10.3389/fphys.2018.00056 (PMC5808183; doi:10.3389/fphys.2018.00056)
Supplement: Supplementary file 6 [file DataSheet1.docx]

Supplementary Material

**Absence of Rapid Propagation through the Purkinje Network as a Potential Cause of Line Block in the Human Heart with Left Bundle Branch Block**

**Jun-ichi Okada^1^, Takumi Washio^1^, Machiko Nakagawa^2^, Masahiro Watanabe^2^, Yoshimasa Kadooka^2^, Taro Kariya^3^, Hiroshi Yamashita^3^, Yoko Yamada^4^, Shin-ichi Momomura^4^, Ryozo Nagai^3^, Toshiaki Hisada^1^, Seiryo Sugiura^1^**

*** Correspondence:** Jun-ichi Okada: [okada@sml.k.u-tokyo.ac.jp](mailto:okada@sml.k.u-tokyo.ac.jp)

# Supplementary Information

As shown in Supplementary Fig. S1, we defined three domains in the body, and the propagation of excitation was formulated differently in each of the domains.


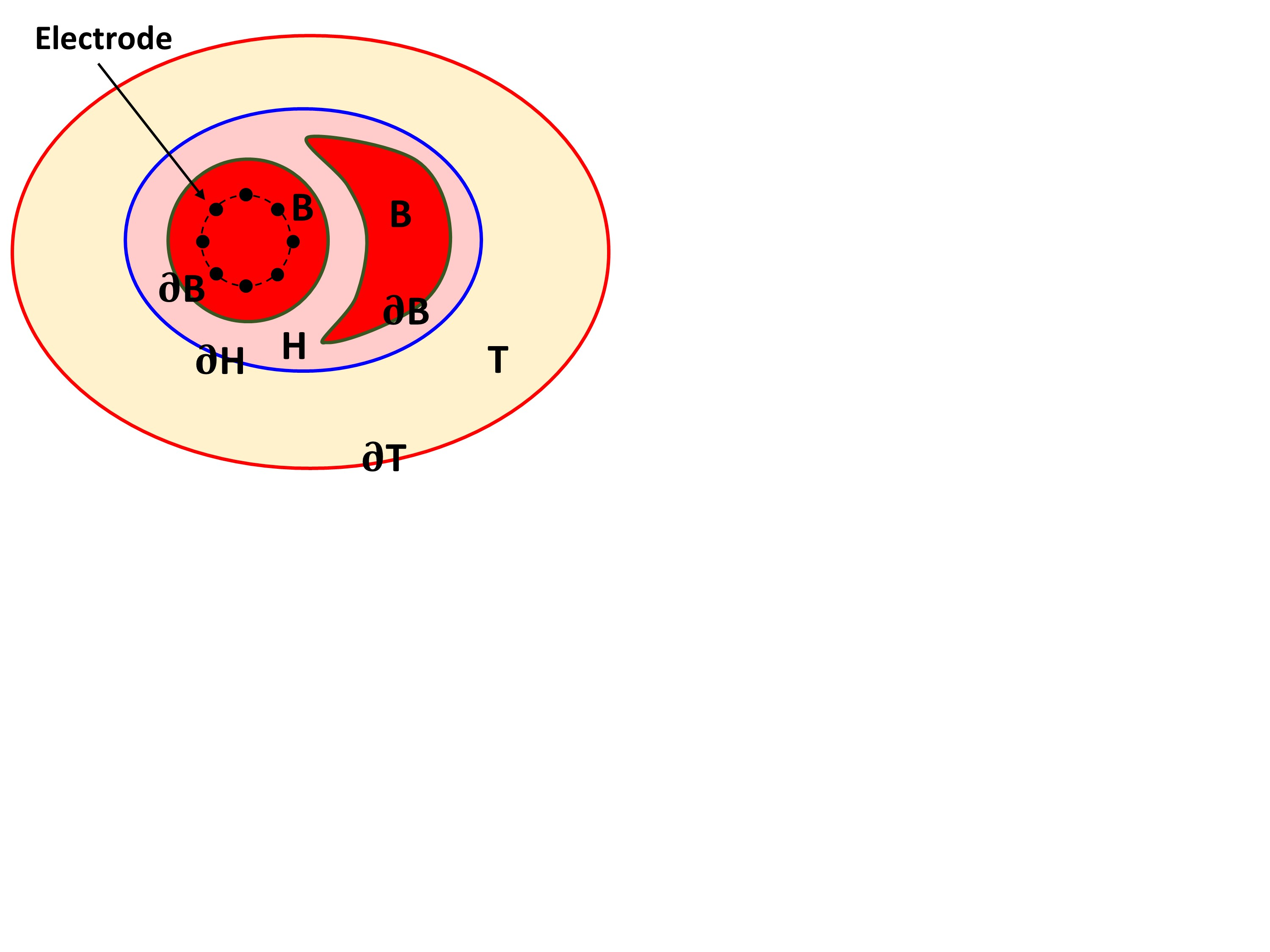


**Supplementary Fig. S1.** Domains in the model. H: heart domain; B: blood domain; T: tissue domain; $\partial H$: boundary of heart domain; $\partial B$: boundary of blood domain; $\partial T$: boundary of tissue domain

As shown in the results section (2.1), the excitable behavior of cardiac tissue (domain H) was modeled as a continuous system using the following bidomain equations:

| $\beta\left( C_{m}\frac{\partial V}{\partial t}+I_{ion} \right)=I_{stim}-\frac{\partial}{\partial x_{i}}\left( G_{ij}^{E}\frac{\partial\emptyset^{E}}{\partial x_{j}} \right)$ | (S1) |
| --- | --- |
| $\beta\left( C_{m}\frac{\partial V}{\partial t}+I_{ion} \right)=I_{stim}+\frac{\partial}{\partial x_{i}}\left( G_{ij}^{I}\frac{\partial\emptyset^{I}}{\partial x_{j}} \right),$ | (S2) |

where $\emptyset^{E}$ and $\emptyset^{I}$ are the extracellular and intracellular potentials, respectively, V = $\emptyset^{I}- \emptyset^{E}$ is the transmembrane voltage, $\beta$ is the surface-to-volume ratio of the tissue, $C_{m}$ is the membrane capacitance, t is time, $G_{ij}^{E}$ and $G_{ij}^{I}$ are the extra- and intracellular anisotropic conductivity tensors, respectively, $I_{stim}$ is the stimulation current, $I_{ion}$ is the sum of ionic currents calculated by the cellular model of electrophysiology, and indices $i$ and $j$ vary from 1 to 3. In the torso domain (T) and blood (B) domains, Laplace’s equation was solved. On the boundaries of each domain, the following conditions were imposed including the electrical coupling between the extracellular space of the heart domain and the torso domain ($\partial H)$ or the blood domain $\left( \partial B \right).$

| $n_{i}\left( G_{ij}^{T}\frac{\partial\emptyset^{T}}{\partial x_{j}} \right)=0, on \partial T,$ | (S3) |
| --- | --- |
| $n_{i}\left( G_{ij}^{I}\frac{\partial\emptyset^{I}}{\partial x_{j}} \right)=0, on \partial H,$ | (S4) |
| $n_{i}\left( G_{ij}^{E}\frac{\partial\emptyset^{E}}{\partial x_{j}} \right)=n_{i}\left( G_{ij}^{T}\frac{\partial\emptyset^{T}}{\partial x_{j}} \right), \emptyset^{E}=\emptyset^{T}, on \partial H,$ | (S5) |
| $n_{i}\left( G_{ij}^{E}\frac{\partial\emptyset^{E}}{\partial x_{j}} \right)=n_{i}\left( G_{ij}^{B}\frac{\partial\emptyset^{B}}{\partial x_{j}} \right), \emptyset^{E}=\emptyset^{B}, on \partial B.$ | (S6) |

By applying the divergence theorem to the weak form of the bidomain equations, we obtain:

| $\int_{H} \delta\emptyset^{I}\beta\left( C_{m}\frac{\partial V_{m}}{\partial t}+I_{ion} \right)dV=-\int_{H} G_{ij}^{I}\frac{\partial\delta\emptyset^{I}}{\partial x_{i}}\frac{\partial\emptyset^{I}}{\partial x_{j}}dV+\int_{\delta H} \delta\emptyset^{I}n_{i}G_{ij}^{I}\frac{\partial\emptyset^{I}}{\partial x_{j}}dS,$ | (S7) |
| --- | --- |
| $\int_{H} \delta\emptyset^{E}\beta\left( C_{m}\frac{\partial V_{m}}{\partial t}+I_{ion} \right)dV=\int_{H} G_{ij}^{E}\frac{\partial\delta\emptyset^{E}}{\partial x_{i}}\frac{\partial\emptyset^{E}}{\partial x_{j}}dV-\int_{\delta H} \delta\emptyset^{E}n_{i}G_{ij}^{E}\frac{\partial\emptyset^{E}}{\partial x_{j}}dS.$ | (S8) |

Note the second term of the right-hand side of (S7) becomes zero from (S4). The finite element discretization of (S7) and (S8) leads to the following matrix representation.

| $\beta\left\{ I_{m} \right\}=-\left[ K_{I} \right]\left\{ \emptyset_{I} \right\},$ | (S9) |
| --- | --- |
| $\beta\left\{ I_{m} \right\}=-\left\{ F_{E} \right\}+\left[ K_{E} \right]\left\{ \emptyset_{E} \right\}.$ | (S10) |

By subtracting (S9) from (S10) and with the relationship $\{V_{m}\}={\{\emptyset}_{I}\}-\{\emptyset_{E}\}$, we obtain:

| $\left[ \left[ K_{I} \right]+\left[ K_{E} \right] \right]\left\{ \emptyset_{E} \right\}=\left\{ F_{E} \right\}-\left[ K_{I} \right]\left\{ V_{m} \right\}.$ | (S11) |
| --- | --- |

Laplace’s equations in the torso and blood domains are discretized to give:

| $\left[ K_{T} \right]\left\{ \emptyset_{T} \right\}=\left\{ F_{T} \right\},$ | (S12) |
| --- | --- |
| $\left[ K_{B} \right]\left\{ \emptyset_{B} \right\}=\left\{ F_{B} \right\}.$ | (S13) |

From (S3), (S5) and (S6), the fluxes and potentials on each boundary satisfy the following conditions:

| $\left\{ F_{E} \right\}=\left\{ F_{T} \right\} , \left\{ \emptyset_{E} \right\}=\left\{ \emptyset_{T} \right\}, on \partial H,$ | (S14) |
| --- | --- |
| $\left\{ F_{E} \right\}=\left\{ F_{B} \right\} , \left\{ \emptyset_{E} \right\}=\left\{ \emptyset_{B} \right\}, on \partial B,$ | (S15) |
| $\left\{ F_{T} \right\}=0, on \partial T.$ | (S16) |

In the finite element method, the continuity of the potential and the conservation of nodal current are satisfied by sharing the degrees of freedom on the boundary. Finally, the matrix form describing the whole system is obtained as:

| $\left[ \begin{matrix} \left[ K_{I} \right]+\left[ K_{E} \right] & \left[ K_{I} \right]+\left[ K_{E} \right] & \left[ K_{I} \right]+\left[ K_{E} \right] & 0 & 0 \\ \left[ K_{I} \right]+\left[ K_{E} \right] & \left[ K_{I} \right]+\left[ K_{E} \right]+\left[ K_{T} \right] & 0 & \left[ K_{T} \right] & 0 \\ \left[ K_{I} \right]+\left[ K_{E} \right] & 0 & \left[ K_{I} \right]+\left[ K_{E} \right]+\left[ K_{B} \right] & 0 & \left[ K_{B} \right] \\ 0 & \left[ K_{T} \right] & 0 & \left[ K_{T} \right] & 0 \\ 0 & 0 & \left[ K_{B} \right] & 0 & \left[ K_{B} \right] \end{matrix} \right]\left\{ \begin{matrix} \emptyset_{E in H} \\ \emptyset_{E,T on \partial H} \\ \emptyset_{E,B on \partial B} \\ \emptyset_{T in T} \\ \emptyset_{B in B} \end{matrix} \right\}=\left\{ \begin{matrix} -\left[ K_{I} \right]\left\{ V_{m} \right\} \\ -\left[ K_{I} \right]\left\{ V_{m} \right\} \\ -\left[ K_{I} \right]\left\{ V_{m} \right\} \\ 0 \\ 0 \end{matrix} \right\},$ | (S17) |
| --- | --- |

The algorithm for time evolution is shown in Supplementary Fig. S2. A summary of the model definition is provided in Supplementary Table S1.


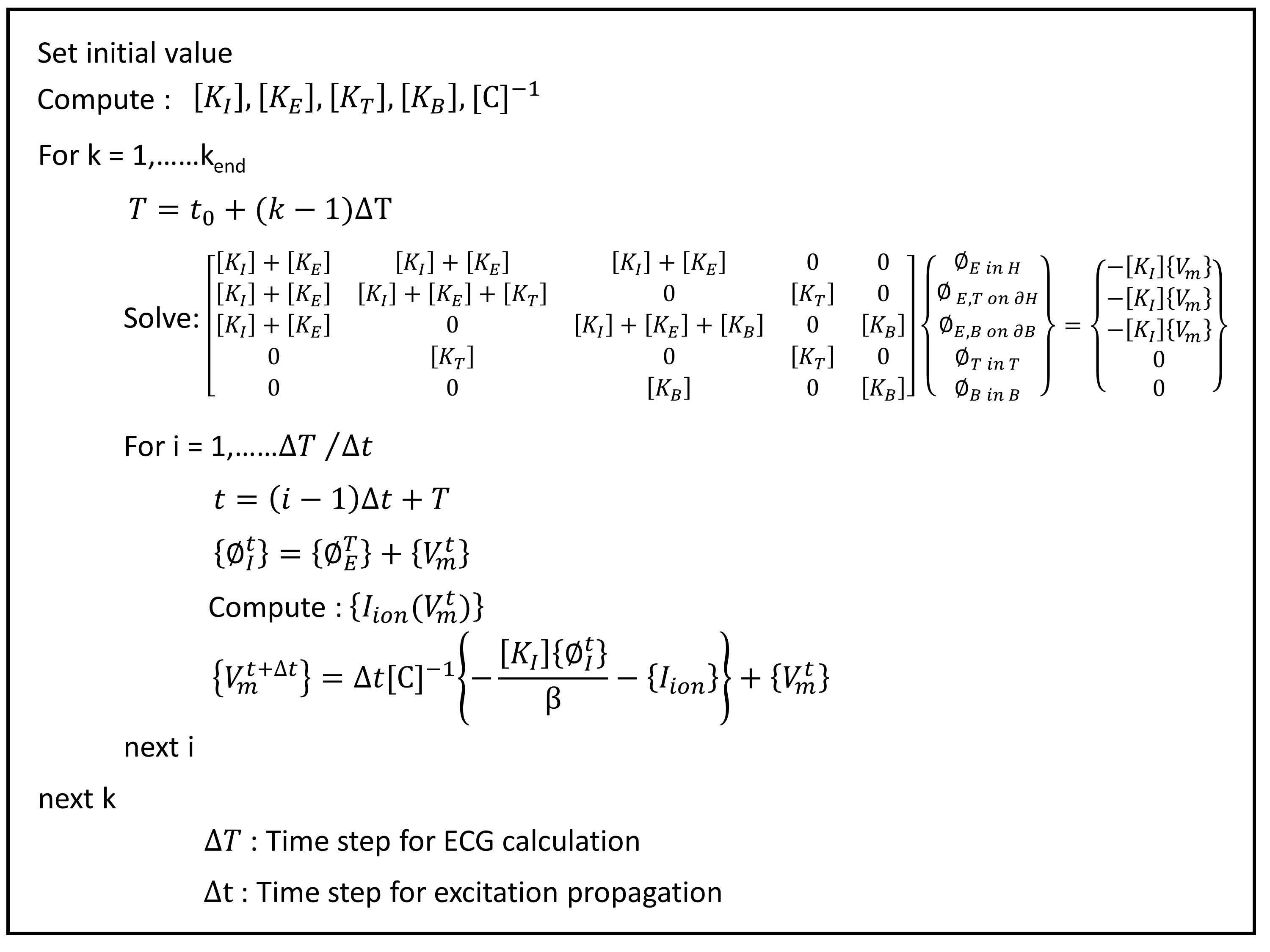


**Supplementary Fig. S2.** Calculation algorithm.

**Supplementary Table S1.** Model parameters and model definition.

| Time step for ECG calculation [ms] | 1 |
| --- | --- |
| Time step for excitation propagation [ms] | 0.01 |
| Mesh size of heart region [cm] | 0.04 |
| Mesh size of torso region [cm] | 0.16 |
| $\beta$: Surface area to volume ratio [$\mathrm{cm}^{-1}$] | 2000 |
| $C_{m}$: Membrane capacitance $\left[ {\mu F}/{\mathrm{cm}^{2}} \right]$ | 1 |
| Element type | Eight-node hexahedral elements |
| Basis function | Bi-linear |
| Pre-conditioners | Incomplete LU |
| Matrix solver | Generalized minimal residual |
| System architecture | Distributed memory |

# Supplementary movies

**Supplementary movie S1:** Membrane potential (left) and non-contact endocardial map (right) during rapid propagation through the Purkinje network.

**Supplementary movie S2:** Membrane potential (left) and non-contact endocardial map (right) during slow propagation without the Purkinje network under the intrinsic rhythm.

**Supplementary movie S3:** Membrane potential (left) and non-contact endocardial map (right) during slow propagation without the Purkinje network under pacing.

**Supplementary movie S4:** Membrane potential (left) and non-contact endocardial map (right) when the Purkinje network was removed from the heart with rapid propagation.

**Supplementary movie S5:** Membrane potential (left) and non-contact endocardial map (right) when the Purkinje network was added to the heart with slow propagation.
